# Leucine and ACE inhibitors as therapies for sarcopenia (LACE trial): study protocol for a randomised controlled trial

**DOI:** 10.1186/s13063-017-2390-9

**Published:** 2018-01-04

**Authors:** Margaret M. Band, Deepa Sumukadas, Allan D. Struthers, Alison Avenell, Peter T. Donnan, Paul R. Kemp, Karen T. Smith, Cheryl L. Hume, Adrian Hapca, Miles D. Witham

**Affiliations:** 1 Tayside Clinical Trials Unit, University of Dundee/NHS Tayside, Dundee, UK; 2 Division of Molecular and Clinical Medicine, University of Dundee, Dundee, UK; 3 Health Services Research Unit, University of Aberdeen, Aberdeen, UK; 4 Population Health Sciences Division, University of Dundee, Dundee, UK; 5 Molecular Medicine, National Heart and Lung Institute, Imperial College, London, UK; 6 School of Medicine, University of Dundee, Ninewells Hospital, Dundee, DD1 9SY UK

**Keywords:** Older people, Perindopril, Leucine, Sarcopenia, Physical function, Quality of life, Randomised controlled trial

## Abstract

**Background:**

Sarcopenia (the age-related loss of muscle mass and function) is a major contributor to loss of mobility, falls, loss of independence, morbidity and mortality in older people. Although resistance training is effective in preventing and reversing sarcopenia, many older people are sedentary and either cannot or do not want to exercise. This trial examines the efficacy of supplementation with the amino acid leucine and/or angiotensin converting enzyme inhibition to potentially improve muscle mass and function in people with sarcopenia. Promising preliminary data exist from small studies for both interventions, but neither has yet been tested in adequately powered randomised trials in patients with sarcopenia.

**Methods:**

Leucine and ACE inhibitors in sarcopenia (LACE) is a multicentre, masked, placebo-controlled, 2 × 2 factorial randomised trial evaluating the efficacy of leucine and perindopril (angiotensin converting enzyme inhibitor (ACEi)) in patients with sarcopenia. The trial will recruit 440 patients from primary and secondary care services across the UK. Male and female patients aged 70 years and over with sarcopenia as defined by the European Working Group on Sarcopenia (based on low total skeletal muscle mass on bioimpedance analysis and either low gait speed or low handgrip strength) will be eligible for participation. Participants will be excluded if they have a contraindication to, or are already taking, an ACEi, angiotensin receptor blocker or leucine. The primary clinical outcome for the trial is the between-group difference in the Short Physical Performance Battery score at all points between baseline and 12 months. Secondary outcomes include appendicular muscle mass measured using dual-energy X-ray absorptiometry, muscle strength, activities of daily living, quality of life, activity using pedometer step counts and falls. Participants, clinical teams, outcomes assessors and trial analysts are masked to treatment allocation. A panel of biomarkers including microRNAs, neurohormones, genetic polymorphisms and markers of inflammation relevant to muscle pathophysiology will be measured to explore predictors of response and further elucidate mechanisms underlying sarcopenia. Participants will receive a total of 12 months of either perindopril or placebo and either leucine or placebo.

**Discussion:**

The results will provide the first robust test of the overall clinical and cost-effectiveness of these novel therapies for older patients with sarcopenia.

**Trial registration:**

ISRCTN, ISRCTN90094835. Registered on 18 February 2015.

**Electronic supplementary material:**

The online version of this article (doi:10.1186/s13063-017-2390-9) contains supplementary material, which is available to authorized users.

## Background

Sarcopenia (the age-related loss of muscle mass and function) is a major contributor to loss of mobility, falls, loss of independence, morbidity and mortality in older people [1]. The mechanisms behind the development of sarcopenia are not fully understood, but accumulating evidence suggests that the condition is multifactorial in aetiology. Some of the factors involved are an altered turnover of muscle protein with a reduction in muscle protein synthesis and a relative increase in muscle protein breakdown, chronic inflammation with increased cytokines including TNF and IL-6, inactivity, mitochondrial dysfunction and altered neuromuscular junction structure and function [2]. Currently, the intervention with the most evidence for efficacy in preventing and reversing sarcopenia is resistance exercise training [3]. However, many older people are sedentary, and either cannot exercise or do not want to exercise.

Non-exercise interventions to prevent or counter the effects of sarcopenia are thus required. A range of potential interventions have been proposed, including protein supplementation, myostatin inhibitors, testosterone and selective androgen receptor modifiers, growth hormone and novel interventions including activin ligands [2]. Many of these approaches have either suffered from frequent side effects (e.g. testosterone, growth hormone) or are under evaluation in early-phase studies by the pharmaceutical industry. The LACE trial studies the efficacy of two promising interventions (leucine and angiotensin converting enzyme inhibition) that can potentially improve muscle mass and function in people with sarcopenia as defined by the European Working Group on Sarcopenia (EWGSOP) [4]. Promising preliminary data exist from small trials for both interventions, but neither has yet been tested in adequately powered trials enrolling people with established sarcopenia.

### Potential benefits of leucine therapy

Muscle protein synthesis in response to protein ingestion is attenuated in older people compared to younger people (i.e. there is anabolic resistance to protein supplementation) [5]. Increasing the amount of protein ingested is one way of overcoming this issue, but older, frail people typically already have sub-optimal protein intakes and increasing their protein intake may be challenging in practice.

Leucine, a branched-chain amino acid, is known to have important regulatory actions, mediated at least in part via the mammalian target of rapamycin (mTOR) pathway. Leucine affects protein turnover, both by reducing proteolysis and enhancing protein synthesis in vitro. Studies in healthy older people confirm that addition of leucine to a protein meal enhances muscle protein synthesis [6–8]; previous studies suggest that approximately 2.5 g of leucine per meal is sufficient to generate the effect [9]. In addition, leucine stimulates insulin release by pancreatic beta cells [10]; insulin signalling not only improves glucose uptake by skeletal muscle, but is also an important anabolic signal for skeletal muscle.

### Potential benefits of ACE inhibitor therapy

The renin–angiotensin–aldosterone system activity has been implicated in skeletal muscle dysfunction via multiple biological pathways [11]. Angiotensin II impairs endothelial function and hence muscle blood supply, is associated with increased levels of inflammation and suppression of IGF-1, and has important effects on mitochondrial function [12–14]. Aldosterone also has deleterious effects, including lowering serum potassium, impairment of endothelial function and promotion of fibrosis [15]. Conversely, ACEi drugs have been shown to have multiple potentially beneficial effects on skeletal muscle function. ACEi drugs improve endothelial function and angiogenesis and reduce inflammation. They improve mitochondrial function, enhance IGF-1 levels, promote skeletal muscle glucose uptake [16] and suppress pro-inflammatory cytokine levels such as IL-6 [17] thought to have important effects on skeletal muscle.

### Existing trial evidence

Leucine-enriched amino acid supplements have been shown to improve muscle strength and function in healthy older people, although one study of leucine alone failed to replicate this effect [18, 19]. However, no studies to date have examined the effect of leucine supplementation alone specifically in people with diagnosed sarcopenia. This is precisely the group, with lower IGF-1 signalling, lower protein intake and poor anabolic response to protein loading, who are most likely to demonstrate a response to intervention. Small trials of protein loading in frail older people show improvements in physical performance measured using the Short Physical Performance Battery test (1.0-point improvement at 24 weeks) [20] but no improvement in skeletal muscle mass over this short follow-up period. In contrast, in older participants undergoing resistance training, protein supplementation enhanced muscle mass, but not muscle strength or performance compared to placebo [21], suggesting that it is the non-exercising majority of older, frail people who are most likely to benefit from nutritional efforts to enhance muscle protein synthesis.

There is some evidence that multiple biological functions of ACEi drugs may translate into clinical benefit. The ACEi perindopril produces a significant improvement in physical function (31-m improvement in 6-minute walk; improvement in quality of life of 0.09 points on the EQ5D tool) in functionally impaired older people with a mean age 79 years [22]. Observational studies report better muscle strength and larger lower extremity mass in older people taking an ACEi [23, 24]. However, studies of ACEi use in fitter older people have not shown positive results [25], which suggests that the effects of ACEi drugs may be more relevant in frailer people. Similar to studies with leucine, combining an ACEi with exercise training also had no effects beyond those evident from exercise training; no additional improvement in 6-minute walk was seen with perindopril in our previous trial in which all participants were undertaking an exercise programme [26]. Thus, the existing evidence suggests that frail older people not engaged in exercise training are the target population most likely to benefit from ACEi therapy. ACEi drugs have not yet been studied specifically in people with sarcopenia who have both poor muscle mass and function, and providing evidence of efficacy in this group with a well-defined specific deficit in muscle physiology is key to testing whether ACEi drugs really have clinically relevant benefit in the target population for this commissioned call (i.e. people with sarcopenia).

### Why the LACE trial is needed now

Sarcopenia underlies two of the most important medical syndromes affecting older people—falls and immobility. It is a major underlying cause of physical frailty in older people, and is associated with a range of adverse outcomes, including increased need for care, increased risk of admission to care home, prolonged hospital stay and increased mortality. As such, sarcopenia is a major cause of morbidity in older people; hence, ways of preventing or treating sarcopenia need to be tested.

To date, few large trials have been conducted of treatments in trial populations selected explicitly to have sarcopenia. Thus our knowledge of how best to conduct such trials, as well as our knowledge of the best treatments to use, is limited. It is against this background that the Efficacy and Mechanisms Evaluation programme, funded by the UK National Institute for Healthcare Research and the UK Medical Research Council, released a commissioning call in 2013 which led to the funding of this trial.

A key problem in pharmacotherapy for older people is the issue of polypharmacy. The use of increasing numbers of different medications (often to treat several co-existing diseases) is a major cause of drug–drug interactions and adverse events—which potentially outweigh any benefit of adding new drugs. The choice of perindopril and leucine as agents to test in this trial was informed in part by the opportunity to use an agent present in the diet already with a low risk of side-effects (leucine) and a medication already commonly used in older people to treat conditions including heart failure and hypertension (perindopril). Using agents with multiple beneficial effects across a range of disease states (pleiotropic agents) is one potential way to maximise the benefit of pharmacotherapy in older, multimorbid patients whist avoiding the pitfalls of polypharmacy.

### Trial objectives

The primary objective of the LACE trial is to determine the efficacy of leucine and perindopril in improving physical function in older people with sarcopenia diagnosed using the EWGSOP definition. Secondary objectives are to evaluate the effect of leucine and perindopril on muscle mass in older people and to evaluate biomarkers that can predict response to leucine and perindopril in patients with sarcopenia.

## Methods

### Study design

LACE is a multicentre, placebo-controlled, 2 × 2 factorial randomised controlled trial analysed as randomised. Participants will be randomised to receive either perindopril or placebo, plus leucine or placebo. The intervention and follow up will be for 1 year. Participants, clinical teams, outcomes assessors and trial analysts are masked to treatment allocation.

### Study population

The study will recruit 440 community-dwelling participants aged 70 and over with sarcopenia according to the EWGSOP definition [4]. Inclusion and exclusion criteria are presented in Table 1, and the criteria for diagnosing sarcopenia in the trial are presented in Table 2.

**Table 1:** Inclusion and exclusion criteria for the LACE trial

Inclusion criteria
Age 70 and over
Diagnosis of sarcopenia (see Table 2)
Exclusion criteria
Contraindications or existing indications to therapies or placebo
Known clinical diagnosis of chronic heart failure (by European Society of Cardiology criteria)
Confirmed LV systolic dysfunction on any imaging modality
Known aortic stenosis (peak gradient > 30 mmHg)
Systolic BP < 90 mmHg (supine)
Dizziness on standing associated with a postural drop of > 20/10 mmHg (asymptomatic orthostatic hypotension is not a contraindication)
Serum creatinine > 170 μmol/L or eGFR < 30 ml/min by MDRD4 calculation
Serum potassium > 5.0 mmol/L
Serum sodium < 130 mmol/L
Using ACEi, angiotensin receptor blocker, aldosterone blocker or leucine already (protein supplementation is permitted)
Previous adverse reaction to ACEi or leucine
Current use of oral NSAIDs (aspirin is permitted, as are topical NSAIDs)
Current use of potassium supplements, aliskiren, spironolactone or other potassium-sparing diuretics
Hereditary or idiopathic angioedema
Lactose intolerance
Contraindications to consent or undertaking study outcomes
Implantable cardioverter defibrillator or pacemaker with atrial sensing lead (pacemakers with ventricular sensing lead only are allowed)
Peripheral oedema present above knee level
Unable to mobilise without human assistance (walking aids are allowed)
Unable to give written informed consent
Currently enrolled in another intervention research study, or < 30 days since completing another intervention research study. Concomitant enrolment in observational studies is permitted.
Overlap with other myopathic conditions or important confounders
Currently enrolled in a time-limited exercise-based rehabilitation programme
Any progressive neurological or malignant condition with life expectancy < 6 months
Severe chronic obstructive pulmonary disease (GOLD stage IV)
Known myositis or other established myopathy
Self-reported weight loss of > 10% in last 6 months (to exclude significant cachexia)
Known uncontrolled thyrotoxicosis
Prednisolone use ≥ 7.5 mg/day (or equivalent dose of other glucocorticoids)

*ACEi* angiotensin converting enzyme inhibitor, *BP* blood pressure, *eGFR* estimated glomerular filtration rate, *GOLD* Global initiative for chronic Obstructive Lung Disease, *LACE* Leucine and Angiotensin Converting Enzyme inhibitors in sarcopenia, *LV* left ventricular, *MDRD4* Modification of Diet in Renal Disease 4-component equation, *NSAID* non-steroidal anti-inflammatory drug

**Table 2:** Screening thresholds for diagnosis of sarcopenia in the LACE trial

	Males	Females
Walking speed over 4 m (m/s)	< 0.8	< 0.8
Maximum handgrip strength (kg)	< 30	< 20
Height-adjusted skeletal muscle mass:		
BMI < 18.5 (kg/m^2^)	≤ 6.02	≤ 5.25
BMI 18.5–24.9 (kg/m^2^)	≤ 7.14	≤ 5.70
BMI 25.0–29.9 (kg/m^2^)	≤ 8.00	≤ 6.19
BMI ≥ 30 (kg/m^2^)	≤ 8.77	≤ 6.72

Diagnosis requires low walking speed and/or low grip strength AND low muscle mass

*BMI* body mass index, *LACE* Leucine and Angiotensin Converting Enzyme inhibitors in sarcopenia

**Fig. 1:**
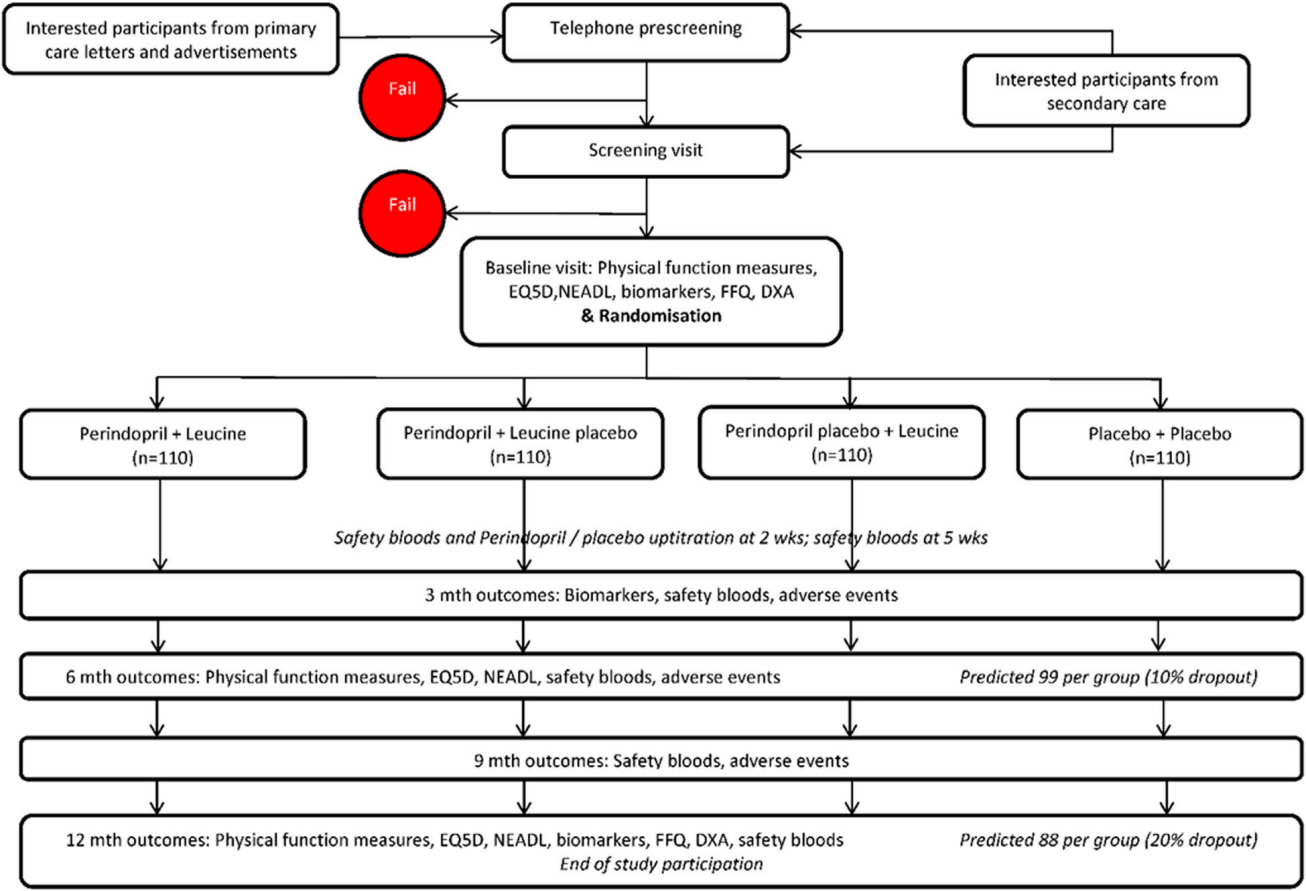
Participant flow through the trial. DXA dual-energy X-ray absorptiometry, EQ5D EuroQol 5D, FFQ Food Frequency Questionnaire, NEADL Nottingham Extended Activities of Daily Living

Potential participants are recruited from both primary care and secondary care clinics, including Medicine for the Elderly clinics, falls clinics and general medicine clinics, at each site. Where local patient registries are kept these are exploited to search for potentially suitable participants. High numbers of patients need to be screened, as patients cannot be identified by diagnosis (sarcopenia is only rarely diagnosed in clinical practice at present) and primary and secondary care electronic systems in the UK do not hold data on physical function that would facilitate identification of those who are more likely to have sarcopenia. A telephone pre-screening phase is therefore included (Fig. 1). This allows rapid, efficient selection of participants most likely to pass a screening visit, and represents the best available strategy for practical screening in sarcopenia trials. Pre-screening is based on a brief telephone conversation conducted by the research nurse exploring any contraindications (e.g. heart failure, taking ACEi) and on administration of the SARC-F tool [27]. The SARC-F tool consists of five questions about physical function, giving a score between 0 (best) and 10 (worst); the score has been developed to assist with screening for sarcopenia and functional impairment. A threshold score is used to denote a high probability of having sarcopenia, and prompts progression to a screening visit. The threshold score was initially set at 4 points or above (out of 10) but is reviewed regularly in the light of screening information, and is adjusted up or down to maximise efficiency of recruitment.

At the screening visit, muscle mass is determined using bioimpedance analysis. This approach minimises the need to perform large numbers of DEXA scans for trial screening, thus reducing costs, enhancing participant convenience and improving recruitment efficiency. The Akern BIA (Bioimpedance Analyser) 101 machine is used at all sites, and appendicular muscle mass is calculated using the equation developed by Sergi et al. [28]; this equation was developed in a white, older European population using the BIA 101 machine. Thresholds for muscle mass are derived from the UK Biobank cohort [29] and are stratified by body mass index for each sex; such an approach ensures that participants with sarcopenic obesity are represented in the trial and is consistent with the more recent Foundation for the National Institutes of Health (FNIH) consensus guideline on diagnosing sarcopenia [30]

Primary care recruitment is carried out with the assistance of the primary care research networks in Scotland (SPCRN) and England (CRN). Network involvement via the Ageing UKCRN specialty group assists with recruitment, access to patients and access to network research nurse time. Written informed consent is obtained from all participants prior to enrolment; the full participation information and consent form are accessible via Additional files 1 and 2. Research ethics committee approval has been given by the East of Scotland Research Ethics Committee (reference 14/ES/1099). The trial is approved by the UK Medicines and Healthcare Regulatory Agency (EudraCT number 2014-003455-61) and the trial is registered online (www.isrctn.com, ISRCTN90094835). The trial is funded by the UK National Institute of Healthcare Research (NIHR) and Medical Research Council (MRC) Efficacy and Mechanisms Evaluation programme (reference 13/53/03). The Sponsor is Tayside Academic Health Sciences Centre, a joint initiative of the University of Dundee and NHS Tayside, and the Sponsor project number for this trial is 2013GR06. Trial management is provided by Tayside Clinical Trials Unit (UKCTU number 49). The protocol on which this report is based is version 7.0, dated 27 April 2017.

### Intervention

Participants are randomised to receive either perindopril 4 mg once daily or matching placebo, and to receive leucine 2.5 g three times per day or matching placebo. The perindopril/placebo is administered as oral capsules. Placebo and active capsules are identical in external appearance. Perindopril tablets are over-encapsulated with a hard gelatin capsule and backfilled with lactose. The placebo capsules are identical in appearance and packed with lactose. The starting dose of perindopril is 2 mg daily which is up-titrated to 4 mg after 2 weeks if tolerated and renal function, serum potassium levels and blood pressure remain within defined limits (potassium < 5.0 mmol/L, creatinine < 180 μmol/L and < 30 μmol/L rise from baseline, systolic blood pressure > 90 mmHg). Participants in the placebo group also undergo a ‘mock’ up-titration. If the 4 mg daily dose is not tolerated, participants are down-titrated to the original 2 mg daily. If 2 mg is also not tolerated, medication is withdrawn.

The leucine/placebo is administered as a powder. A 1.5-ml scoop is provided and participants are instructed to take three scoops (4.5 ml equivalent to 2.5 g of leucine/placebo) three times per day. The placebo is matching lactose powder. Participants are instructed that they should mix the powder with food or drink. Exploratory work with our local Older People’s Advisory Group indicated that mixing the leucine powder with food or drink was palatable, particularly if mixed with foods such as yoghurt or orange juice.

Randomisation is conducted via a centrally controlled, web-based Good Clinical Practice-compliant randomisation system, run by Tayside Clinical Trials Unit. Investigators at each site access the web-based system to randomise each participant at the end of the baseline visit. Randomisation is stratified by site. To ensure balanced assignment across critical variables, a minimisation algorithm is employed, using baseline age (> 80 vs ≤ 80 years), sex, SPPB score (> 8 vs ≤ 8), grip strength (> 25 vs ≤ 25 kg for males; > 15 vs ≤ 15 kg for females) and Charlson co-morbidity score (> 3 vs ≤ 3) to balance allocation across trial arms.

Participants are asked to return all unused study medication including empty bottles/containers. Adherence to medication will be recorded by capsule count of the perindopril/placebo capsules and by the weight of the container for the leucine/placebo powder. Serum angiotensin converting enzyme (ACE) is measured to assess adherence to the perindopril/placebo capsules. To ensure that medication adherence is maximised, we employ a combination of written information about the study medication and why taking it is important, together with aide memoirs including LACE-branded mugs and fridge magnets. At each visit, participants are reminded about the importance of medication adherence. Interventions incorporating these components have been shown in a Cochrane review to enhance adherence [31]. If one of the study drugs needs to be stopped or the participant wishes to stop, they are encouraged to continue with the other study drug. Participants stopping both study drugs are encouraged to remain in the study in order to facilitate analysis as randomised.

### Outcomes

**Fig. 2:**
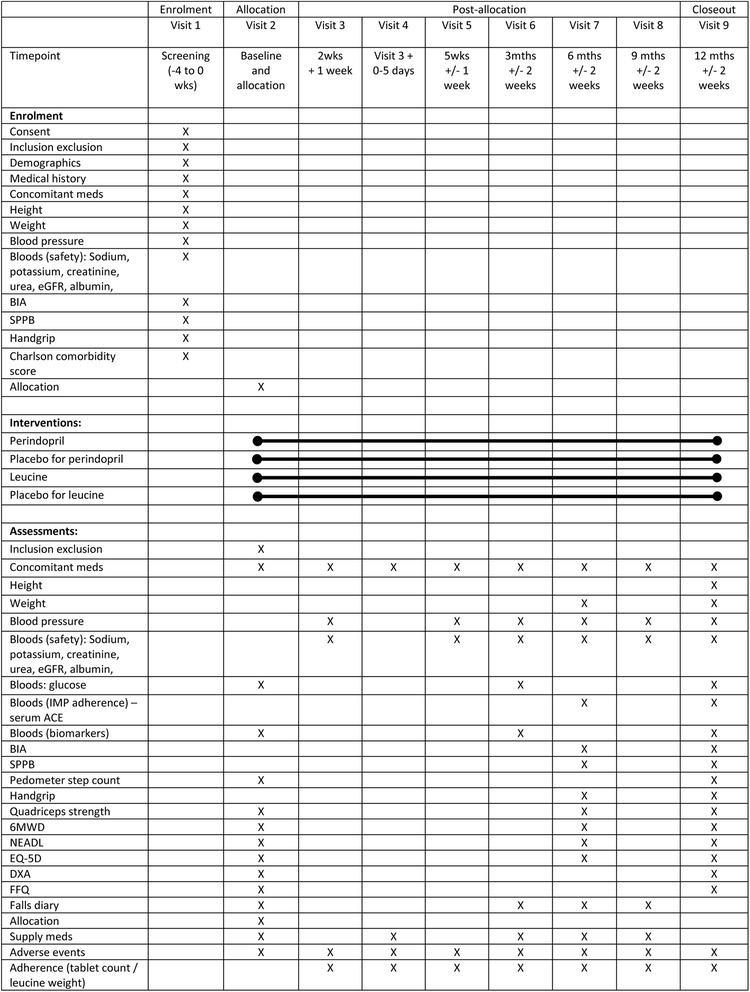
SPIRIT diagram for the LACE trial. 6MWD six minute walking distance, ACE angiotensin converting enzyme, BIA bioimpedance analysis, DXA dual-energy X-ray absorptiometry, eGFR estimated glomerular filtration rate, EQ5D EuroQol 5D, FFQ Food Frequency Questionnaire, IMP investigational medicinal product, NEADL Nottingham Extended Activities of Daily Living, SPPB Short Physical Performance Battery

The primary outcome for the trial is the Short Physical Performance Battery, measured at baseline, 3, 6, 9 and 12 months. The between-group difference in the SPPB across the whole follow up will be reported as the primary outcome, calculated using generalised estimating equations to incorporate repeated measures and missing data. The SPPB is a validated measure of lower limb function that reflects everyday activity. It is a powerful predictor of subsequent disability, institutionalisation and death in older people [32–34], and has more data validating its use in older people than any other measure of physical function. Secondary outcomes include between-group differences in muscle mass measured by dual X-ray absorptiometry, other measures of physical function, instrumental activities of daily living and quality of life. We also measure blood pressure, insulin resistance, hip bone mineral density and a panel of biomarkers to explore both mechanisms of disease and predictors of response to treatment. Key outcomes and times of collection are listed as part of the SPIRIT figure (Fig. 2).

In addition, we are collecting data on adverse events, falls, hospitalisation, death and admission to institutional care. Consent is obtained for open-label follow up of participants for these outcomes collected from routine clinical data following the end of the 1-year study period. We are collecting data on usual physical activity and diet at baseline and 12 months using step counts measured by an OMRON pedometer and the Scottish Collaborative Group Food Frequency Questionnaire (FFQ) respectively; this will allow the interaction between treatment and protein intake (total, plant vs animal protein) to be ascertained.

### Statistical analysis

Analyses will be by group as randomised and will comply with the International Conference on Harmonisation (ICH) E9 ‘Statistical Principles for Clinical Trials’ [35]. Two-sided *p* < 0.05 will be taken as significant for all analyses. The primary analysis will be a repeated-measures, mixed-model between-group comparison of SPPB utilising all available data points during follow up. Initially, a test for treatment interaction between perindopril and leucine will be carried out and if not significant the main analysis will proceed using the full power of the factorial design; each intervention will be assessed separately as an independent hypothesis. Treatment effect estimates for all primary and secondary outcomes will be adjusted for baseline values, age, sex and minimisation variables.

Secondary outcomes will be analysed with similar methodology using repeated-measures, mixed-model, between-group comparisons. Unadjusted and adjusted analyses will be presented as already discussed. Sensitivity analyses will be performed to further test the effect of missing data both by multiple imputation and by assigning worst-possible result status to missing data points. A supplementary per-protocol analysis will be performed to examine adverse events in those taking at least 80% of their study medication.

No interim analyses are planned. Exploratory sub-group analyses will be performed, including examining differences in treatment effects by age (above and below median), SPPB (> 8 vs ≤ 8) and sex. A full Statistical Analysis Plan will be prepared and finalised prior to trial database lock, and will be made available as an additional upload on the *Trials* website once finalised.

### Biomarkers sub-study

The analytes that will be investigated in the biomarker sub-study can be divided into three main groups; those that reflect the activity of the renin–angiotensin system or its targets, those that are markers of protein turnover and those potential novel markers of regenerative capacity that may reflect the capacity to respond to treatment. To look at the activity of the renin–angiotensin system we will measure circulating levels of ACE and renin as well as two truncated forms of angiotensin (ang(1–7) and ang(1–9)) that have been shown to alter muscle mass and are likely to be modified by an ACEi [36, 37]. There are a number of different processes through which activation of the renin–angiotensin system is thought to contribute to muscle loss, including increased inflammatory cytokine production, reduced IGF-1 production and increased myostatin production in signalling [38]. We will therefore quantify IL-6, TNF-α and myostatin. We will also quantify circulating levels of GDF-15, another member of the TGF-β family of cytokines, as it is a marker of all-cause mortality and is associated with muscle mass in patients with COPD [39] and with muscle loss following surgery [40]. The influence of specific polymorphic variation in the renin–angiotensin system will also be analysed.

To identify changes in muscle protein turnover in the absence of direct measurements, which are expensive and invasive, we will quantify pro-collagen III N-terminal peptide as a measure of new collagen synthesis [41] and levels of circulating muscle restricted miRNAs (myomiRs) that are increased in the circulation of patients with diseases associated with muscle wasting including Duchenne muscular dystrophy [42] and in chronic obstructive pulmonary disease [43].

Finally, we will examine circulating levels of a set of imprinted miRNAs that may reflect the regenerative capacity of the individual. These miRNAs include miR-518e from a paternally expressed cluster on chromosome 19 and miR-485-3p from a maternally expressed cluster on chromosome 14 which we have shown to be associated with muscle phenotype in patients with COPD [44, 45].

Univariate analyses will be conducted examining the interaction between individual baseline biomarker values and treatment effect for muscle mass and the SPPB at 12 months, and between biomarker change between baseline and 3 months and treatment effect for muscle mass and the SPPB at 12 months. In addition, multivariable analysis for baseline values including age, sex, SPPB, grip, muscle mass, albumin, creatinine, all measured biomarkers and treatment group will be conducted to examine whether baseline values or change at 3 months predicts the SPPB change and muscle mass change at 12 months.

### Sample size calculation

We have taken a deliberately conservative approach and used the minimum clinically important difference (MCID) for the SPPB to ensure we have proof of efficacy. In order to detect a MCID in the SPPB of 0.5 points (anticipated SPPB of 8 in the placebo group and 8.5 in the intervention group, SD = 2.7 points [46]) with a power of 90% at α = 0.05, and assuming a correlation between time points of 0.7 as seen from previous work [26], we would require a total of 352 participants (88 for each of the four groups). Assuming 20% dropout at 12 months (a rate of attrition consistent with that seen in previous trials of ACEi in frail older people [26]), we therefore need to recruit 440 patients. This sample size will also have 90% power to detect a 5% difference in muscle mass at 12 months, assuming a baseline value of 19 kg (SD = 2.8) for total skeletal muscle mass [47].

### Trial oversight and monitoring

Trial oversight is provided by an independent Trial Steering Committee (TSC), which meets at least every 6 months. Members include academic geriatricians with trials experience and three lay members, together with the chief investigator. The TSC reviews trial progress, approves any significant protocol changes, advises on trial conduct and approves analysis plans and dissemination plans. The TSC reports to the funder (NIHR), who has final decision-making authority for the trial. An independent Data Monitoring Committee (DMC) meets at least every 6 months; this committee comprises three independent members, chaired by an academic geriatrician with trials experience, and contains a minimum of one independent statistician. The DMC reviews trial adverse events and safety data, and makes recommendations to the TSC. Charters for conduct of the TSC and the DMC are available from the study team and from the funder. Routine management of the trial is provided by the Trial Management Group (TMG), which comprises the grant co-applicants, local site investigators, trials unit personnel from project management, data management and statistics teams, Sponsor representatives and Sponsor monitors.

Monitoring of the trial conduct at each site is provided by the Sponsor. Monitoring takes place according to a pre-specified plan, with site visits conducted according to the recruitment rate and frequency of problems identified by remote monitoring of study data. Both the Sponsor and the Clinical Trials Unit are subject to periodic audit by the UK Medicines and Healthcare products Regulatory Agency. The funder provides monitoring of overall trial progress, with additional input from the independent TSC. The final decision to publish lies with the funder (NIHR) and the Sponsor; the funder and the Sponsor have both reviewed the study design. The Sponsor will have no input into data collection, analysis and interpretation; the final report will be peer reviewed by the Funder prior to finalisation.

### Protocol amendments

Significant protocol amendments are implemented across all sites after approval by the Sponsor, the independent TSC, the funder, the ethics review board, the UK Medicines and Healthcare products Regulatory Agency and the UK Health Research Authority. The current protocol is available via the NIHR website and via the trial website (www.lacetrial.org.uk).

### Data confidentiality and post-trial care

Trial data are held separately from personal identifiers, on password-protected secure servers at the University of Dundee. Identifiable data will not be shared outside the trial team or Sponsor without written participant consent. De-identified trial data will be stored for 15 years after the end of the trial. Supplies of trial medication will not be made available to participants after the end of the trial, but both interventions are available via either prescription or via health shops. Compensation for harm suffered as a result of trial interventions or processes will be available via UK National Health Service compensation or via University of Dundee trial indemnity.

### Authorship and dissemination

Authorship for the main trial results will be based on the International Committee of Medical Journal Editors authorship criteria, and will include, but not be limited to, grant co-applicants, local investigators, trial managers, statisticians and data analysts fulfilling these criteria. Dissemination of results will take place via multiple routes: presentation at scientific conferences; publication in scientific journals and via the NIHR library; presentations to participants and their families; written summaries of results to participants; and presentations to local healthcare teams. Results will be uploaded to the EudraCT and ISRCTN databases.

## Discussion

Initial screening criteria for muscle mass in the trial used the Janssen equation to calculate muscle mass [48], and used conservative thresholds for total skeletal muscle mass: 13 kg for women and 20.5 kg for men. Initial experience with screening found that these criteria were too stringent—only 5% of those screened had eligible skeletal muscle mass. Further investigation suggested that the Janssen equation had systematic inaccuracies at low skeletal muscle mass, and our thresholds did not account for body mass index—thus excluding patients with sarcopenic obesity. Since changing to the screening thresholds described in this article, the screening pass rate now approaches 50% and recruitment has consequently accelerated.

Because sarcopenia is not a diagnosis commonly made in clinical practice (it received an ICD-10 code only in September 2016), case-finding for sarcopenia trials is challenging and few centres in the UK have experience recruiting to trials for sarcopenia. A supplementary aim of the LACE trial programme is therefore to determine efficient ways of finding and screening potential participants for sarcopenia trials. We will evaluate the success of recruitment from primary versus secondary care; the best threshold on the SARC-F to identify eligible participants on telephone pre-screening; the best combination of search terms to use in primary care electronic system searches; and the best ways of engaging participants from a range of secondary care clinics. Initial experience suggests that primary care recruitment is much more successful than recruitment from geriatric medicine secondary care services. Although one might expect patients with sarcopenia to be concentrated in such secondary care clinics, this patient group has multiple exclusion criteria, particularly use of ACEi drugs and the presence of dementia. The overall number of patients in geriatric medicine clinics is small compared to the volume of potential participants in primary care; the lower percentage of eligible patients in primary care makes efficient searches, mailshots and telephone pre-screening essential. We are setting up a centralised telephone facility to manage the large volume of telephone pre-screening required by this approach.

A final aim is to build capacity across a network of UK sites to recruit to and deliver sarcopenia trials. We anticipate that the experience gained by UK sites in the trial, along with other initiatives such as the British Geriatrics Society Sarcopenia and Frailty Research Group [49], will aid in the establishment of this UK Sarcopenia Trials Network. This network, and the expertise developed by centres through LACE, should accelerate clinical trial activity in this emerging and important area of medicine.

### Trial status

The trial started recruitment in July 2016, and results are expected towards the end of 2020.

## Additional files

Additional file 1: Informed consent form for the LACE trial. (PDF 29 kb)

Additional file 2: Participant information leaflet for the LACE trial. (PDF 467 kb)

## Abbreviations

ACE: Angiotensin converting enzyme
ACEi: Angiotensin converting enzyme inhibitor
BIA: bioimpedance analysis
EQ5D: EuroQol 5D
EWGSOP: European Working Group on Sarcopenia in Older People
FFQ: Food Frequency Questionnaire
FNIH: Foundation for the National Institutes of Health
GDF-15: Growth differentiation factor 15
ICH: International Committee on Harmonisation
IGF-1: Insulin-like growth factor 1
IL-6: Interleukin-6
LACE: Leucine and ACE inhibitors
miRNA: Micro RNA
NIHR: National Institute of Healthcare Research
SARC-F: Sarcopenia Function questionnaire
SPPB: Short Physical Performance Battery
TGF: Transforming growth factor
TNF: Tumour necrosis factor
