# Supplementary material for: Leucine and ACE inhibitors as therapies for sarcopenia (LACE trial): study protocol for a randomised controlled trial
Source: Trials. 2018 Jan 4;19:6. doi: 10.1186/s13063-017-2390-9 (PMC5753568; doi:10.1186/s13063-017-2390-9)
Supplement: Supplementary file 1 — Informed consent form for the LACE trial. (PDF 29 kb) [file 13063_2017_2390_MOESM1_ESM.pdf]

LACE

Participant Study ID | | | | | |

PARTICIPANT INFORMED CONSENT FORM

LACE trial

Perindopril and Leucine to improve muscle function in older people

<u>This form must be completed and signed by the research participant in the presence of Principal Investigator or someone from the research team designated by the Principal Investigator.</u>

		Initials
I have read and understood the Participant Information Sheet for the LACE trial Version 4.0, 30-09-2016	Yes ☐No☐	
I have spoken to Dr, Mr. Mrs, Miss<br><br>______________________________	Yes ☐No☐	
I have had the opportunity to discuss the study and to ask questions. All my questions have been answered to my satisfaction.	Yes ☐No☐	
I agree to my GP being informed of my participation in this study.	Yes ☐No☐	
I understand, and I agree, that my identifiable routine blood tests and bone scan results will be stored within the NHS clinical system and will be available to doctors looking after me in the future.	Yes ☐No☐	
I understand that my participation in the study is voluntary and that I am free to leave the study at any time without having to give a reason and that this will not affect my medical care in any way.	Yes ☐No☐	
I understand that relevant sections of my medical notes and data collected during the study may be looked at by the research team or from the regulatory authorities or appropriate staff from the University of Dundee or NHS Tayside or the local NHS Trust, where it is relevant to my taking part in this study. I give permission for these individuals to have access to my records.	Yes ☐No☐	
I agree to be informed of any significant clinical finding found during my participation in the research project and agree that members of the research team can contact both me and my GP and inform any referral specialist required to carry out further investigations.	Yes ☐No☐	
I agree that if I withdraw or I am withdrawn from the study that data already collected can be retained and included in the data analysis.	Yes ☐No☐	

LACE

Participant Study ID ☐☐☐☐☐

I understand and agree that the information and research blood samples that I provide will be gifted by myself, transferred to specialist laboratories and analysed by members of the study team or stored (link-anonymised) for up to 15 years and can be used for future, as yet unspecified, medical research into health, illness and medical treatment. This research will be subject to proper scientific and ethical review.	Yes ☐No☐	☐
I agree that the research team can access my medical records, in both paper and electronic form, to obtain information on my health now and over the next five years to investigate the long-term effects of the study treatments.	Yes ☐No☐	☐
I agree to take part in the above study.	Yes ☐No☐	☐

This research is approved by The East of Scotland Research Ethics Service REC 2

Name of participant	Date	Signature
Name of person taking consent	Date	Signature

1 copy for participant; 1 copy to be filed in the hospital notes; the original to be filed in the ISF.
