# Supplementary material for: Leucine and ACE inhibitors as therapies for sarcopenia (LACE trial): study protocol for a randomised controlled trial
Source: Trials. 2018 Jan 4;19:6. doi: 10.1186/s13063-017-2390-9 (PMC5753568; doi:10.1186/s13063-017-2390-9)
Supplement: Supplementary file 2 — Participant information leaflet for the LACE trial. (PDF 467 kb) [file 13063_2017_2390_MOESM2_ESM.pdf]

LACE

UNIVERSITY OF DUNDEE

LOCAL SITE LOGO

www.lacetrial.org.uk

PARTICIPANT INFORMATION SHEET

The LACE trial

Perindopril and Leucine to improve muscle function in older people

We would like to invite you to participate in a research project which is a collaboration between [LOCAL PI] and Dr Miles Witham at the University of Dundee, Scotland. We believe it to be of potential importance. However, before you decide whether or not to participate, we need to be sure that you understand firstly why we are doing the study, and secondly what it would involve if you agreed to take part. We are therefore providing you with the following information. Please take time to read it carefully and be sure to ask any questions you have, and, if you want, discuss it with others. We will do our best to explain and provide any further information you may ask for now or later. You do not have to make an immediate decision.

Why are we doing this study?

Muscle weakness in older people is common, and can cause problems, such as getting about and doing daily tasks. Muscle weakness also leads to falls in older people, which in turn can lead to broken bones and hospital admissions.

So finding ways to improve muscle strength in older people is really important. At the moment, exercise using weights or elasticated bands is the only treatment that we know works. Not everyone can do such exercise, and not everyone wants to do exercise. And even people who do exercise still lose muscle. We need to find new ways of improving muscle strength in older people, and this study will test two new ways of doing that – a medication called perindopril and a food supplement called leucine.

Why have I been contacted?

We have contacted you because you are aged 70 or over. Whilst not everyone of your age has weakness of their muscles, many do and so this trial may be suitable for you to take part in.

Do I have to take part?

It is up to you to decide. Participation in this study is entirely voluntary and you are free to refuse to take part or to withdraw from the study at any time without having to give a reason and without this affecting your future medical care or your relationship with medical or nursing staff looking after you. If you, Dr [PI] or one of your clinicians decides you should withdraw from the study, we would like your permission to retain and analyse the data already collected. If you do take part, we will also ask for your permission to access your medical records (your notes and electronic records) for the next five years, so that we can see the long-term effects of the study treatments.

What will happen to me if I take part?

If you agree, you will take part in the study for one year. We can pay for a taxi or other transport to get you to the hospital and back for each study visit if this would be helpful.

The telephone call

We will first phone you at home to ask you some short questions about your health, whether you have fallen, and whether you have any difficulty doing everyday activities. If these questions suggest that the study is suitable for you, we will arrange for you to visit your local hospital for a screening visit

The screening visit

At the screening visit, we will go over the study information; you will have the opportunity to ask any questions you want to about the study. If you are happy to take part, we will ask you to sign a consent form.

We will take a note of your medicines and medical conditions, check your blood pressure, height and weight. We will measure your muscle size by attaching some electrodes to your foot and wrist, you do not feel anything while this is done. We will take a blood sample (about a teaspoonful), ask you to walk a few yards, test your grip strength, test your balance and get out of a chair five times whilst we time you. We will let you know at the end of the visit if you are suitable to take part. This visit will take about 1 hour.

The main study

If the screening visit confirms that you are eligible to go into the main trial, we will ask you to come up to the hospital for a slightly longer visit, which will take a couple of hours. This may be done on the same day as the screening visit if you have had recent blood tests done which confirm you are eligible to take part. We will ask you to fast from midnight the night before your visit if your visit is in the morning, if your visit is in the afternoon you may have a light breakfast then fast from then until your visit. You should take your usual medications as normal and we will provide a snack for you. At this visit, we will ask you to do the following:

- Walk as far as you can up and down a corridor for six minutes. You can rest as often as you want when you do this, and you can use a stick or other walking aid if you wish to
- Test your leg strength
- Answer two questionnaires on your quality of life and daily activities

- Give some blood (about three tablespoonsful)
- Have a scan of your muscles and bones. This involves a very small dose of X-rays (less than you would get with a chest X-ray) and does not involve any needles. While we will try and arrange for all of these tests to be done at the one visit, it may be necessary or more convenient for you to have them done on separate days.

At the end of the visit, we will ask you to fill in a questionnaire about what you eat, take home a diary to record any falls you might have over the next three months, and wear a small device (a pedometer or step counter) during the day for a week. This clips onto your waistband, and tells us how far you walk in a day. We will then give you a supply of the two study medications.

Follow-up visits

At two and five weeks and at three and nine months the study nurse will visit you at home or you can come to the hospital, whichever is more convenient, to check your blood pressure, take 1-2 teaspoonfuls of blood, and adjust the dose of your

perindopril. 1-5 days after the visit at two weeks the nurse will deliver your next batch of medication to you at home or you can come to the hospital to collect them. Each visit will take around 15 minutes.

At 6 and 12 months, we will ask you to attend the hospital for study visits. Each visit will take between one and two hours; we will repeat combinations of the tests that you did at the screening and baseline visits. We have shown what happens at each study visit in the diagram in the middle of this brochure. At the 6 and 12 month visits we will ask you to fast from midnight the night before your visit if your visit is in the morning, if your visit is in the afternoon you may have a light breakfast then fast from then until your visit. You should take your usual medications as normal and we will provide a snack for you.

After you finish

We will send you the study's Newsletter which will keep you up-to-date on the study's progress and if you have access to the internet you can log onto the study's website; www.lacetrial.org.uk for

updates. At the end of the study, we will invite you to a tea party to tell you all about the results, and we will send a summary of the results to you as well.

What is being tested?

We are testing whether perindopril or leucine can improve muscle strength and muscle size in older people with muscle weakness. People from across the UK will be taking part; a quarter will receive both treatments, a quarter will receive dummy (placebo) treatments, and the rest will receive one treatment and one dummy treatment. The treatments will be allocated at random, and neither you nor the study team or your doctors will know which you are on until the end of the study.

The perindopril or matching dummy pill is a single capsule that you take once a day (usually in the morning). The leucine or matching dummy comes as a tub of powder. We will ask you to mix 3 small scoops of this with your food or drink at each meal (three times a day). We will provide you with some serving suggestions that other people have

found to work well for taking the leucine – they have all told us that it tastes fine mixed in with things such as yoghurt and orange juice.

Visit Schedule for the study

Visit time	Screening (before start)	Baseline (start)	2 weeks	2 weeks plus 0-5 days	5 weeks	3 months	6 months	9 months	12 months
Informed consent	√								
Medical history & medications	√								
Blood pressure	√		√		√	√	√	√	√
Blood sample	√	√	√		√	√	√	√	√
Test muscle size	√						√		√
Balance, walk and stand test	√						√		√
Pedometer walking count		√							√
Handgrip strength	√						√		√
Leg strength		√					√		√
6 minute walk test		√					√		√
Questionnaires		√					√		√
Bone and muscle scan (DXA)		√							√
Food diary		√							√
Falls diary		√				√	√	√	
Medication supply and count		√		√		√	√	√	

Will taking part in the study affect your usual care?

We will not alter any of your other medication or interfere with your other treatment. You will continue to be seen by your GP and by any hospital clinics that you usually attend. There are a few medications, particularly some blood pressure tablets and some painkillers (such as ibuprofen) that you will not be able to take regularly as they can interact with the study medications. You should remind any doctor prescribing you a new medication that you are on the trial. If you are started on any new medications please inform the research team.

What are the possible discomforts, risks and side effects?

Perindopril can cause a cough, which affects about one in ten people. Uncommonly, it can cause dizziness or kidney problems with increases in blood levels of potassium. We will monitor your blood tests and blood pressure to catch any problems that might occur. If you think you have taken more capsules than required (overdose) please let the research team know as soon as

possible. Leucine is used as food supplement and is not known to cause any medical problems. Having blood taken may cause minor bruising and discomfort. We will allow you to have plenty of rest in between the walking and strength tests so that you are not made overly tired by the tests.
As part of the trial you will receive two DXA scans (to look at muscle and bone) which you would not normally receive as part of routine care. During the DXA scan the X-ray unit will be above you, and is very similar to having a normal X-ray taken. The DXA scan will expose you to a small amount of radiation similar to the amount the average person is exposed to in 10 days from natural sources in the environment.

What are the benefits of taking part in the study?

You will be monitored closely during the study by the study team. The tests will give us information about the function of your kidneys, your fitness and general wellbeing. If any of these investigations reveal any new abnormality we will either discuss this with your GP (with your consent) or refer you to a specialist clinic [HOSPITAL] (whichever seems most appropriate.)

The study may not immediately benefit you, but might make you feel stronger. If the results of the study are positive this may change how we treat people with muscle weakness just like you.

Will my GP know about this research project?

With your permission we will inform your GP of your participation, any clinical results, and of any new medical problem we find as a result of your participation in the study.

Will my taking part in this study be kept confidential?

All the information that is collected about you during the course of this study will be kept strictly confidential. There will be two sets of information obtained during the study. One set will be routine blood tests analysed by local NHS laboratories and your DXA scan results, and the other, the research data obtained from research blood samples and study procedures. The routine blood test results and DXA scan results obtained will be stored indefinitely using your name and unique hospital

record number within the NHS clinical system and can be made available to specialist doctors for your future health care needs.

Your research data will be stored using a unique study code which is non-identifiable. All written information will be kept in a locked filing cabinet in a locked room. Any web-based data will be stored in a secure password protected central database at Health Informatics Centre, University of Dundee. Only individuals directly involved with the study will have access to this information. It is a requirement of the regulators that your records in this study, together with any other relevant medical records, be made available for scrutiny by appropriate monitors from Tayside Medical Science Centre (TASC) and the Regulatory Authorities. This procedure is routine and carried out by fully qualified officials, and data confidentiality is preserved

At the end of the study the confidential records will be kept for 15 years and then destroyed. The confidential handling, processing, storage and disposal of data are in accordance with the Data Protection Act 1998.

The research blood tests will be analysed by our colleague Dr Paul Kemp in his laboratory in London. Some of the tests will include tests for differences in genes that control muscle function. After the tests are complete, we will, with your permission, store the spare blood samples so that we can perform further tests on them in future as we learn more about muscle function in older people.

Will I continue to receive the medication used in this study after it finishes?

Not usually. The study gives an indication of possible benefit from the medicines being tested and it may be some time before we are sure about how useful it actually is. Unless you need the medication for a different reason, you would not receive either medication after the end of the study.

What will happen to the results?

The results will be examined by the researchers who have organised the study and a short report will be produced. You will not be identified in this report. The results will be shared with the funder for the study (The National Institute for Health Research). The results will then be published in scientific journals. Again, you will not be identified in any journal articles. If you would like the results of the study please inform the research team.

Who is organising and funding this research?

The study has been organised by Dr Miles Witham

and colleagues at the Universities of Dundee, Aberdeen and Imperial College London. This project is funded by the Efficacy and Mechanism Evaluation (EME) Programme, a Medical Research Council (MRC) and National Institute of Health Research (NIHR) partnership.

What are my rights?

If you have a complaint about your participation in the study you should first talk to a researcher involved in your care. You can ask to speak to a senior member of the research team or the Complaints Officer for NHS [RELEVANT BOARD/TRUST].

Complaints and Claims Manager

[LOCAL CONTACT DETAILS]

In the event that something goes wrong and you are harmed during the study there are no special compensation arrangements. If you are harmed and this is due to someone's negligence then you may have grounds for a legal action for compensation against the University of Dundee or

NHS [RELEVANT TRUST] but you may have to pay your legal costs. The normal National Health Service complaints mechanisms will still be available to you (if appropriate.)

Who has reviewed the study?

The East of Scotland Research Ethics Service REC 2 which has responsibility for scrutinising proposals for medical research on humans, has examined the proposal and has raised no objections from the point of view of medical ethics.

For further information contact:

Research Nurse: [name for each centre] on telephone [local number] and [e-mail address].

Principal Investigator: [name for each centre] on telephone [local number] and [e-mail address].

If during the study you become unwell or are concerned, as well as the usual services provided by the NHS such as NHS 111 (Tel: 111), you can also contact the trial team during normal working hours on [CONTACT NUMBER] if you are unwell and need urgent advice or assistance do not delay in seeking further advice or treatment as usual through the NHS services.

Thank you for reading this information sheet and considering taking part in this study. If you would like more information or want to ask questions about the study please contact the study team on the number/addresses above.

University of Dundee

NHS National Institute for Health Research
